# Effects of Dual Targeting of Tumor Cells and Stroma in Human Glioblastoma Xenografts with a Tyrosine Kinase Inhibitor against c-MET and VEGFR2

**DOI:** 10.1371/journal.pone.0058262

**Published:** 2013-03-04

**Authors:** Anna C. Navis, Annika Bourgonje, Pieter Wesseling, Alan Wright, Wiljan Hendriks, Kiek Verrijp, Jeroen A. W. M. van der Laak, Arend Heerschap, William P. J. Leenders

**Affiliations:** 1 Department of Pathology, Radboud University Nijmegen Medical Centre, Nijmegen, The Netherlands; 2 Department of Cell Biology, Nijmegen Centre for Molecular Life Sciences, Radboud University, Nijmegen, The Netherlands; 3 Department of Pathology, VU University Medical Centre, Amsterdam, The Netherlands; 4 Department of Radiology, Radboud University Nijmegen Medical Centre, Nijmegen, The Netherlands; University of Pécs Medical School, Hungary

## Abstract

Anti-angiogenic treatment of glioblastoma with Vascular Endothelial Growth Factor (VEGF)- or VEGF Receptor 2 (VEGFR2) inhibitors normalizes tumor vessels, resulting in a profound radiologic response and improved quality of life. This approach however does not halt tumor progression by diffuse infiltration, as this phenotype is less angiogenesis dependent. Combined inhibition of angiogenesis and diffuse infiltrative growth would therefore be a more effective treatment approach in these tumors. The HGF/c-MET axis is important in both angiogenesis and cell migration in several tumor types including glioma. We therefore analyzed the effects of the c-MET- and VEGFR2 tyrosine kinase inhibitor cabozantinib (XL184, Exelixis) on c-MET positive orthotopic E98 glioblastoma xenografts, which routinely present with angiogenesis-dependent areas of tumor growth, as well as diffuse infiltrative growth. In *in vitro* cultures of E98 cells, cabozantinib effectively inhibited c-MET phosphorylation, concomitant with inhibitory effects on AKT and ERK1/2 phosphorylation, and cell proliferation and migration. VEGFR2 activation in endothelial cells was also effectively inhibited *in vitro*. Treatment of BALB/c nu/nu mice carrying orthotopic E98 xenografts resulted in a significant increase in overall survival. Cabozantinib effectively inhibited angiogenesis, resulting in increased hypoxia in angiogenesis-dependent tumor areas, and induced vessel normalization. Yet, tumors ultimately escaped cabozantinib therapy by diffuse infiltrative outgrowth via vessel co-option. Of importance, in contrast to the results from *in vitro* experiments, *in vivo* blockade of c-MET activation was incomplete, possibly due to multiple factors including restoration of the blood-brain barrier resulting from cabozantinib-induced VEGFR2 inhibition. In conclusion, cabozantinib is a promising therapy for c-MET positive glioma, but improving delivery of the drug to the tumor and/or the surrounding tissue may be needed for full activity.

## Introduction

Glioblastoma is a highly aggressive primary brain tumor that is characterized by extensive areas in which tumor cells diffusely infiltrate the brain parenchyma. A well-known hallmark of this cancer type is the presence of a necrotic core, surrounded by a rim in which hypoxia-induced neovascularization occurs [1]. Angiogenesis in these areas is associated with vessel leakiness, which contributes to edema and high intracranial pressure, aggravating symptoms that by themselves can be lethal. Local vessel leakage is exploited to diagnose glioblastoma, as it results in extravasation of intravenously administered contrast agents like Gd-DTPA which can be readily visualized by MRI. Glioblastomas are generally operated upon to the maximum feasible extent, followed by radiotherapy and chemotherapy with temozolomide. Remnants of diffusely growing tumor cells will however inevitably result in tumor recurrence and median survival is currently still only 14.6 months [2].

It is well recognized now that inhibition of VEGF-A signaling pathways in neovascular endothelial cells, either by the neutralizing antibody bevacizumab or selective VEGFR2 tyrosine kinase inhibitors, induces a radiological response, significantly reduces edema and may substantially improve quality of life [3]–[6]. Bevacizumab is now approved by the FDA for treatment of recurrent glioma. However, it has also become clear from a number of preclinical but also clinical studies that the diffuse infiltrative phenotype of glioblastomas is not sensitive to angiogenesis inhibition [5], [7]–[9]. We previously showed that different anti-angiogenic treatments of orthotopic E98 xenografts (displaying both angiogenesis and diffuse infiltration [10]) affect only the angiogenic tumor component [8], [9], [11]. Apparently, anti-angiogenic therapies drive tumor cells to adapt a resistant, angiogenesis-independent phenotype in which tumor cells obtain their blood supply entirely from pre-existent vasculature [12]–[16]. These therapies have even been suggested to increase tumor cell invasion in glioma and other tumor types [17], [18] and this appears to be associated with induction of hypoxia [19]. It is therefore of major importance for effective glioma treatment that approaches become available that tackle diffuse infiltrative tumor growth.

The c-MET tyrosine kinase receptor has been linked to both tumor angiogenesis and the invasive phenotype of glial and other tumors [19], [20]. Upon binding of its ligand hepatocyte growth factor (HGF, scatter factor), c-MET is phosphorylated on tyrosine residues Y1234/1235 (kinase domain) and Y1349 and Y1356, the latter two residues with their surrounding amino acids functioning as docking sites for substrates such as Gab1, Grb2 and phosphatidylinositol 3 kinase (PI3K) [21], [22]. Downstream signalling of c-MET involves important pathways including RAS/PI3K and ERK/MAPK, which are associated with tumorigenesis and cancer progression [23].

Amplification of the c-MET gene (located on chromosome 7) is seen in glioblastomas [24] and both c-MET and HGF are frequently overexpressed in glioma specimens and cell lines. HGF is a strong stimulator of *in vitro* glioma cell migration [25]–[27] and c-MET expression has also been demonstrated in invasive glioma cells [25]. Simultaneous targeting of the VEGF and c-MET pathways may therefore be an interesting therapeutic approach for c-MET-positive glioblastoma because it will reduce vessel leakage (resulting in edema reduction) and simultaneously may reduce tumor cell migration and thus tumor progression. Cabozantinib (XL-184, Exelixis, South San Francisco, CA) is a small compound tyrosine kinase inhibitor of VEGFR2, c-MET and RET and has been shown to block tumor development in the RipTAG2 mouse model of pancreatic carcinogenesis more effectively than blockade of c-MET or VEGFR2 alone [19], [28], [29]. Cabozantinib was recently FDA-approved for treatment of medullary thyroid cancer.

The aim of the current work was therefore to test the effects of cabozantinib in mice carrying highly aggressive orthotopic E98 glioma xenografts [10]. We show that cabozantinib blocks vascular leakage in this c-MET positive tumor model and gives a significant survival benefit which was not observed in previous experiments from our lab with bevacizumab or other VEGFR2 inhibitors [8], [9]. Interestingly, whereas cabozantinib completely blocked c-MET tyrosine phosphorylation *in vitro* in E98 cell cultures, phosphorylated c-MET was still present in remaining diffuse infiltrative tumor areas in treated mice. We propose that the anti-VEGFR2 activity of cabozantinib results in a restoration of the blood-brain barrier, thereby precluding an efficient distribution of the compound to the tumor cells and reducing c-MET inhibitory activity.

## Materials and Methods

### Cell Culture

E98NT cells (cell line derived from E98 xenografts, described in [30]) were cultured in DMEM supplemented with 10% FCS and pen/strep in the presence of 5% CO_2_ at 37°C. HUVECs were cultured in EBM medium, supplemented with FCS, bFGF and VEGF (Lonza) according to the manufacturer’s instructions.

### Protein Expression Analysis

E98NT cells or HUVECs were plated in 6 well dishes in appropriate media at a density of 5×10^5^ cells per well. The following day cabozantinib (0, 0.01, 0.1, 0.5, 1 and 10 µM in DMSO) was added. After a 30 min incubation, cells were washed with ice-cold PBS and protein extracts were generated using RIPA buffer supplemented with protease and phosphatase inhibitors (Roche Applied Science). Similarly, extracts were generated from E98NT cells treated for 24 h with 1 and 10 µM cabozantinib (for assaying apoptosis) and sections of snap frozen xenograft brains (approximately 20 mg of tissue). Protein lysates were cleared by centrifugation and protein concentrations were measured using the BCA Protein Assay Kit (Thermo Scientific) according to manufacturer’s instructions. Proteins (20–40 µg/lane) were subjected to SDS-PAGE and Western Blotting using antibodies directed against c-MET (clone EP1454Y, Epitomics and clone D1C2, Cell Signaling Technology (CST)), phospho-c-MET (Y1234/1235, clone D26, CST), phospho-AKT (S473, clone D9E, CST) phospho-ERK1/2 (Thr202/Tyr204, clone 20G11, CST), anti-U1-70 (to detect apoptosis, [31]) and α-tubulin (clone 236-10501, Molecular Probes) or γ-tubulin (clone C20, goat, Santa Cruz Biotechnology) as internal control. Antibodies were detected using appropriate secondary antibodies, labeled with IRDye700 or IRDye800 infrared dyes. Signals were visualized and quantified when appropriate, using the Odyssey Infrared Imaging System (LI-COR Biosciences Odyssey Application Software version 3.0.30).

### Dose Response Analysis

IC_50_ of cabozantinib was determined as follows: cells were plated at a concentration of 2×10^4^ cells per well in 96-wells plates. The next day increasing concentrations of cabozantinib (in DMSO) were added to the medium. Each condition was tested in quadruplicate in at least three independent experiments. Metabolic activity of the cells was determined 4 days following start of TKI treatment by incubation with 0.5 mg/ml MTT in PBS (Sigma-Aldrich, St. Louis, MO). After a 3.5 hr incubation at 37°C formazan crystals were dissolved in MTT solvent (0.1% NP40 and 3.4 mM HCl in isopropanol) and optical densities were measured at 560 nm. IC_50_ concentrations were determined using sigmoidal dose-response (variable slope) statistics and normalized in GraphPad Prism.

### Cell Migration Assays

E98 spheroids were generated by the hanging drop method. In brief, 0.7 ml methylcellulose (Sigma M6385, final concentration 1.68 mg/ml) was added to 4.3 ml E98 cell suspension in normal culture medium (500,000 cells total) and drops of 25 µl containing approximately 2,500 E98 cells were seeded in a dry culture dish. The dish was then inverted and incubated overnight at 37°C in the presence of 5% CO_2_. The next day, individual spheroids were seeded in a matrigel-coated 96-well imaging culture dish (Matrigel: BD cat 356237, 96-well plates: BD falcon imaging plates cat 353219) and grown further at 37°C and 5% CO_2._ Individual spheroids were photographed at t = 0 h in a culture system which allows for live cell imaging, after which they were incubated with 0, 1 or 10 µM cabozantinib. After 24 h, wells were washed with PBS and cells were fixed with 4% PFA in 0.1 M phosphate buffer, followed by DAPI staining. Fluorescent and phase-contrast images were then taken and Image J software was used to quantify spheroid outgrowth. Briefly, cells which had migrated from the spheroids were selected automatically using a cell mask. Numbers of cells that had migrated out of the spheroids (n = at least 32 for each condition) were measured. Statistical analyses involved ANOVA and post-hoc Tukey's Multiple Comparison Test.

### Animals

Athymic female BALB/c nu/nu mice (18–25 gram, age 6–8 weeks) were kept under specified pathogen free conditions and received food and water *ad libitum*. The local Animal Experimental Committee of the Radboud University Nijmegen Medical Center approved all experiments. E98 glioblastoma cells derived from subcutaneous xenograft tumors were injected intracranially under isoflurane anesthesia as described previously [10]. All efforts were made to minimize suffering. Animals were closely monitored by visual inspection and weighed daily from start of treatment (see below) and sacrificed when evident signs of tumor burden (especially weight loss >20%, severe neurological dysfunction) were observed. Brain, liver and kidneys were harvested, and parts were formalin fixed and paraffin embedded or snap-frozen in liquid nitrogen for further analysis.

### Therapy

Animals carrying E98 tumors were randomly divided into placebo and cabozantinib treatment groups. Treatment was started at day 12, when signs of tumor growth became apparent, as evidenced by the presence of edema in T2 weighted MR imaging. Water-suspended cabozantinib (XL184, Exelixis, South San Francisco) was given daily by oral gavage in doses of either 60 mg/kg (n = 3) or 100 mg/kg (n = 10) in a volume of 100 µl. A control group (n = 10) received water daily by oral gavage.

### Immunohistochemistry (IHC)

Immunohistochemical stainings were performed as described before [9]. In short, after epitope retrieval by boiling in citrate buffer (pH 6.0), 4 µm tissue sections were incubated with primary antibodies against GLUT1 (Neomarkers), CD34 (clone MEC14.7, Hycult biotech), c-MET (clone EP1454Y, Epitomics), phospho-c-MET (Y1234/1235, clone D26, CST), MCT4 (clone H90, Santa Cruz), cleaved caspase 3A (clone C92-605, BD Pharmingen) and Ki-67 (clone Sp6, Thermo Fisher Scientific). Appropriate biotinylated secondary antibodies were used for detection using the Avidine Biotine Complex (ABC) method (Vector Laboratories). Specific signals were visualized by staining with 3-amino-9-ethyl-carbazole (AEC, Scytek Laboratories) or 3,3′-diaminobenzidine (Power-DAB, ImmunoLogic) solution. All sections were counterstained with haematoxylin and mounted in Imsol Mounting medium (Klinipath B.V.).

### Image Analysis

Cell proliferation, hypoxia and vessel densities were quantified using KS400 software (Carl Zeiss AG, Germany) with a custom-written macro on images acquired on a Zeiss Axioskop II microscope coupled to a CCD-RGB camera. For proliferation, five random non-overlapping diffuse infiltrative or compact tumor containing microscopic fields (magnification x200) were analyzed per section. Compact and diffuse areas were recognized by gross histology, with compact areas being present in the ventricles or leptomeninges, and diffuse areas defined by the intermittent presence of white matter tracts in H&E staining. The proliferative fraction was defined as the number of proliferative cells (based on Ki-67 positivity) divided by the total number of nuclei in a tumor region. Ratios were determined and the average value for the different fields per slide was used in further calculations.

For hypoxia quantification, compact tumor areas, defined as sharply bordered regions lacking normal brain parenchyma in between the tumor cells, and hypoxic regions (based on MCT4 positivity) were selected interactively. The hypoxic fraction was defined as the total hypoxic area divided by the total tumor area.

Vessel densities were measured in GLUT-1 immunostainings and counted in 5 high power fields per section in both compact and diffuse areas. Since neovasculature in compact areas often lack blood-brain barrier characteristics (e.g. GLUT-1 expression), also CD34 stainings were performed.

Statistical comparisons were done by normality analysis, followed by a Student’s t-test (two-sided) using GraphPad Prism v4. For both control and 100 mg/kg cabozantinib treated brains, n = 10. A p value of ≤0.05 was considered as statistically significant.

### Contrast-enhanced Magnetic Resonance Imaging

CE-MRI was performed in a 7T MR system (ClinScan, Bruker BioSpin, Ettlingen, Germany) equipped with a clinical user interface (syngo MR, Siemens, Erlangen, Germany). When tumor-related symptoms became apparent, animals were anesthetized using 1–2% isourane in a 70% N_2_O and 30% O_2_ mixture and placed in a prone position in an MR cradle. Breathing was monitored throughout the MR experiment and the animals’ core temperature was maintained at 37.5°C using a continuous flow of warm air (SA Instruments, Inc., Sunny Brook, NY, USA). Anatomical references were acquired using a multi-slice localizer with slices in the three main orthogonal directions. A turbo spin echo sequence with following settings were used: repetition time (TR) 3880 ms, echo time (TE) 43 ms and a turbo factor of 7. The scans had a resolution of 98 µm in-plane and a thickness of 0.7 mm per slice. A bolus of 0.2 ml of Gd-DTPA (20 mM, Magnevist®, Schering, Germany) was injected intravenously via a pre-inserted tail vein catheter and additional sets of T1-weighted images were acquired 2–3 minutes after injection.

## Results

Elevated expression of c-MET in glioma specimens has been documented by several groups. Whereas up to 35% of primary gliomas overexpress this RTK, this percentage increases to up to 75% in recurrent glioblastomas [32]. Often, heterogeneity in c-MET expression is found between tumor cells [33] and some papers have reported on the expression of c-MET on endothelial cells too [34]. This is especially interesting since multi-targeted tyrosine kinase inhibitors such as cabozantinib have been developed that have specificity against both VEGFR2 and c-MET.

**Figure 1:**
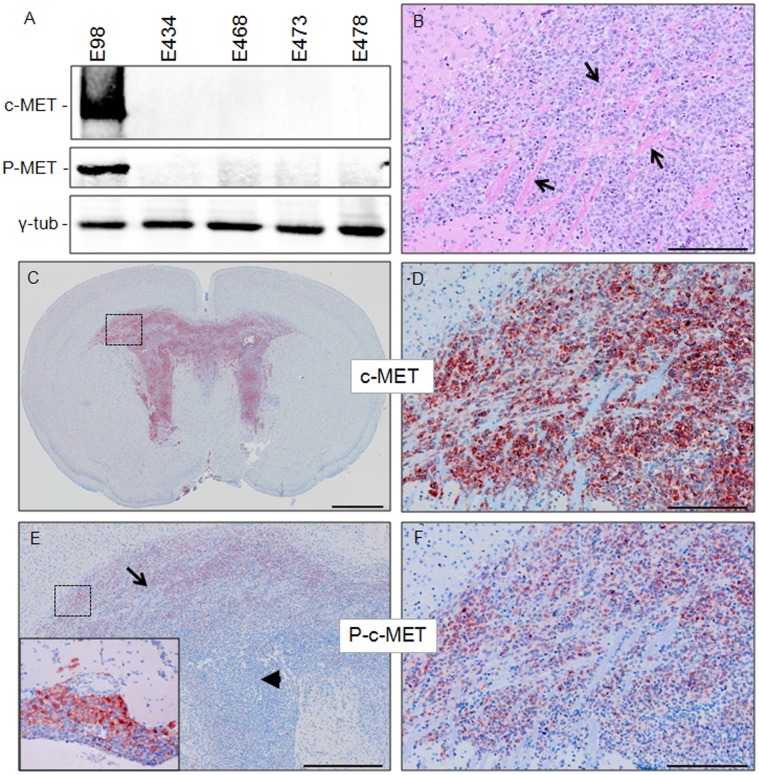
c-MET is activated in E98 xenografts. Panel A shows a Western blot containing protein extracts of different xenografts as indicated (40 µg/lane) and stained with a pan and an Y1234/1235 phosphorylated (P-) c-MET specific antibody. As a loading control, γ-tubulin was included. Immunohistochemical analysis reveals prominent c-MET expression and activation in orthotopic E98 xenografts (C–F). Gross appearances of an E98 tumor are shown in C and E, while D and F show magnifications of the boxed areas in C and E. The H&E section in B illustrates the diffuse nature of these tumors, arrows pointing at white matter tracts and comparison with D shows homogeneous expression of c-MET by tumor cells. Arrow in E points at diffuse infiltrative tumor cells in white matter with activated c-MET, while the arrowhead points at a more compact paraventricular tumor area. The inset in E represents an area with compact leptomeningeal growth partly positive for activated c-MET. The pictures shown are representative for this xenograft model. Size bars: B, D, F 200 µm; C 1 mm and E 500 µm.

In the past we have established a number of glioblastoma xenograft lines by either subcutaneous or direct intracerebral implantation of surgically derived tumor cell suspensions in immunodeficient mice, which are subsequently maintained by serial transplantation [10]. One xenograft line that has been extensively analyzed for its response to anti-angiogenic treatment is E98. This line routinely presents with diffuse tumor growth in the brain parenchyma, using co-opted blood vessels and white matter tracts as scaffold. The presence of additional areas of angiogenesis-dependent growth makes this model of high translational relevance for testing of targeted therapies. Western blot analysis using c-MET and phospho-c-MET specific antibodies (recognizing the phospho-tyrosines Y1234 and Y1235) revealed that only E98 xenografts express significant amounts of the activated receptor, consistent with our observation that chromosome 7 is amplified in this xenograft line (Figure 1A and [10]). IHC analysis confirmed c-MET expression in E98, showing expression in all tumor cells (note that the non-stained structures in Figure 1D correspond to white matter tracts, as is clear from H&E staining of the serial section in Figure 1B). IHC using the phospho-specific anti-c-MET antibody also confirmed the presence of activated c-MET, although in a heterogeneous fashion, being present predominantly in diffuse infiltrating tumor cells in the corpus callosum and adjacent white matter (Figure 1E, arrow and 1F). Furthermore, activated c-MET was detected in a rim of non-infiltrative tumor cells (routinely present in this model in the leptominges and ventricles) at the interface with normal brain parenchyma (inset in Figure 1E and not shown). In central regions of compact growing tumor areas c-MET was not activated (arrowhead in Figure 1E).

**Figure 2:**
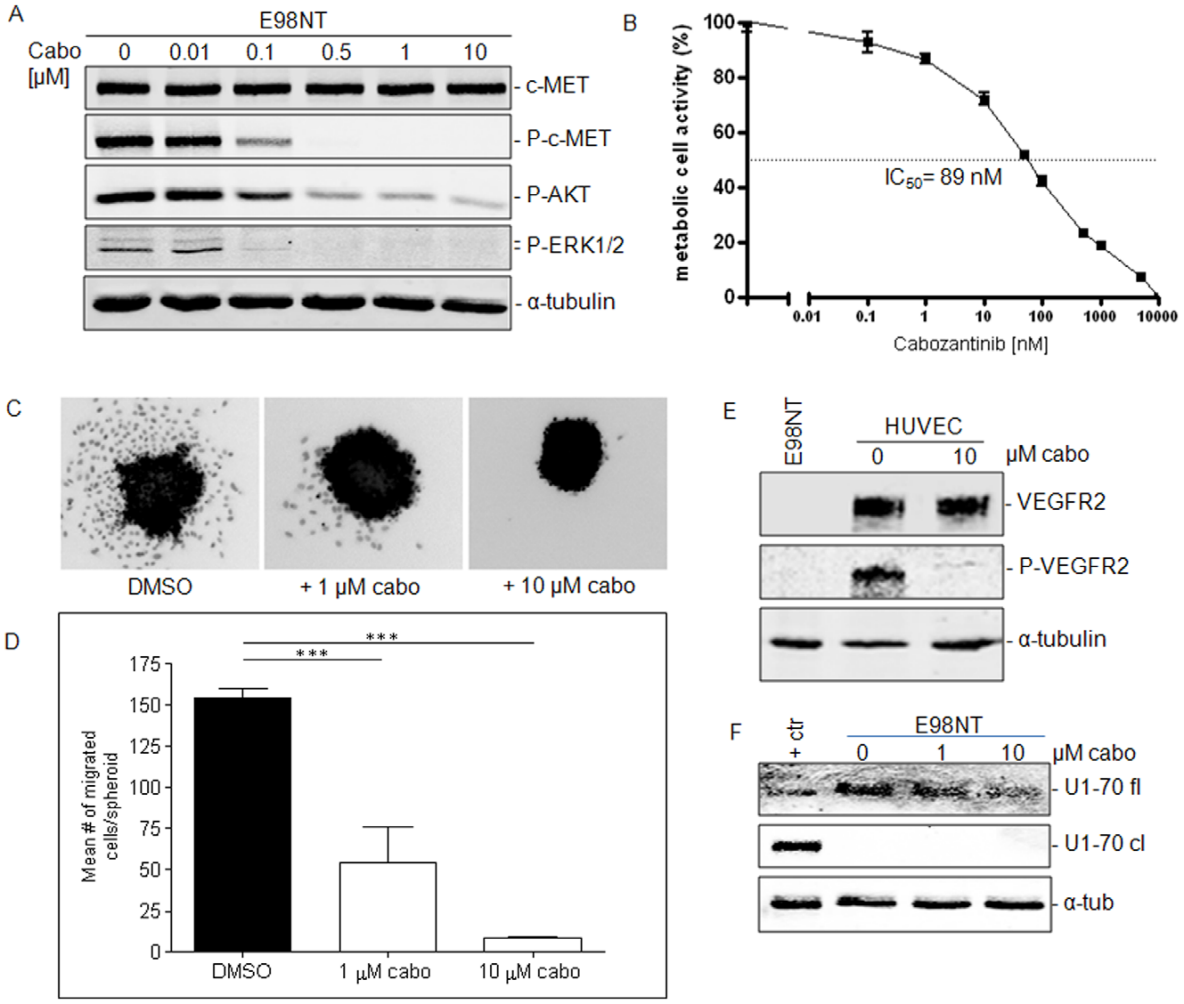
*In vitro* effects of Cabozantinib on c-MET and VEGFR2 signaling. Panel A shows a Western blot of E98NT cell extracts (20 µg per lane) treated for 30 minutes with different concentrations of cabozantinib as indicated. Protein extracts were analyzed for c-MET, phospho-c-MET, AKT, and ERK1/2, using α-tubulin as a loading control. B) MTT assays were done to determine the IC_50_ concentration of cabozantinib on E98NT cells. Experiments were performed at least in triplicate. C) Effects of cabozantinib on cell migration. Shown are representative examples of DAPI-stained spheroids after 24 hr incubation with indicated concentrations. Number of outgrowing and migrating cells per spheroid are shown in panel D (***: p<0.001). Number of migrating cells were significantly different between groups (one-way ANOVA, p<0.0001). Post-hoc Tukey's Multiple Comparison Test revealed significant differences groups as indicated (***: p<0.001). E) Western blot of cell lysates of E98NT and HUVEC extracts, treated with 10 ng/ml VEGF with or without cabozantinib, and stained for VEGFR2, phospho-VEGFR2 and α-tubulin as an internal control. Note the absence of VEGFR2 in E98NT cells. F) Western blot of treated E98NT cell extracts with the anti-apoptotic antibody U1-70. Control sample consists of Jurkat cells treated with anisomycin.

We reasoned that the partially angiogenic character of the E98 xenograft model, in combination with high c-MET expression and activation in diffuse infiltrative areas makes this model highly relevant to study simultaneous inhibition of VEGFR2 and c-MET signaling. First, we investigated whether cabozantinib therapy blocked c-MET activation *in vitro*. Treatment of the E98NT cell line, derived from the E98 xenograft model [30] resulted in an efficient and dose-dependent inhibition of c-MET phosphorylation after 30 minutes (Figure 2A). Downstream signaling via AKT was also significantly inhibited by cabozantinib (note the ∼82% reduction of phosphorylated AKT and the accompanying decrease in phosphorylated ERK1/2 at concentrations higher than 0.5 µM). Consistently, cabozantinib caused a dose-dependent inhibition of proliferation in E98NT cells (Figure 2B, IC_50_ ∼ 89 nM). Cabozantinib did not induce apoptosis *in vitro* as demonstrated by Western blot staining with anti-U1-70 antibody (Figure 2F). In an *in vitro* spheroid-based cell migration assay, we observed that cabozantinib significantly reduced the number of E98 cells that are able to migrate away from the spheroids (Figure 2C–D, p<0.001, Post-hoc Tukey’s Multiple Comparison Test). Thus, c-MET signals have bearing for E98 tumor cell migratory potential as well. These inhibitory effects can be attributed to c-MET inhibition since E98 cells do not express VEGFR2 (Figure 2E). The inhibitory activity of cabozantinib on VEGFR2 [28] was confirmed on cultures of HUVECs and was complete at concentrations of 10 µM (Figure 2E).

**Figure 3:**
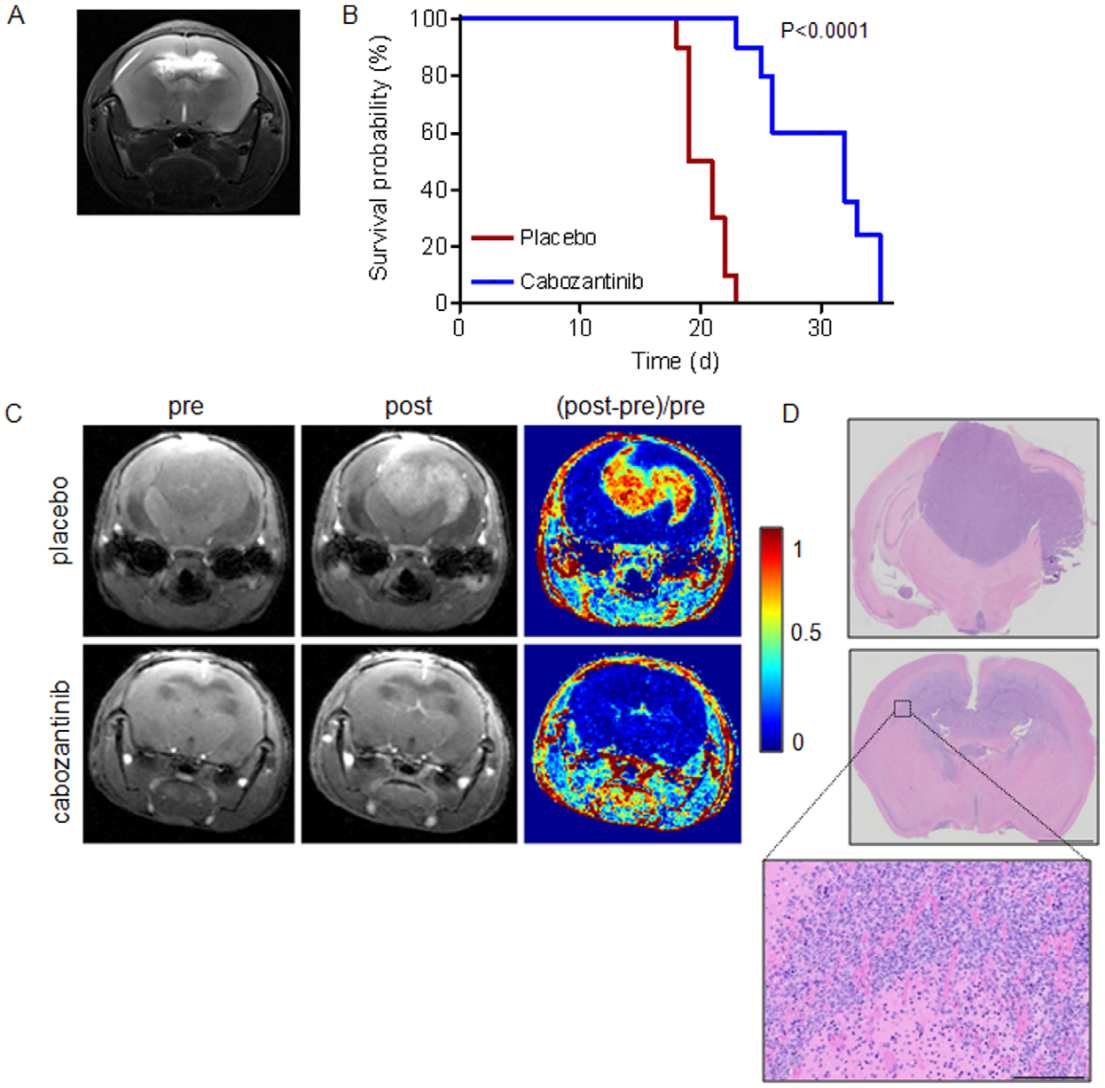
Cabozantinib prolongs survival of mice bearing orthotopic E98- xenografts. Mice were treated with 100 mg/kg cabozantinib from day 12 post tumor inoculation, when tumor was detected via abnormalities in T2 images (see panel A for a representative example). B) Survival curves for placebo (n = 10) and cabozantinib (n = 10) treated animals. Note that, for ethical reasons, mice were sacrificed when excessive weight loss and signs of neurological dysfunction occurred. Median survival was significantly different between the groups (20 and 32 days respectively, log rank test, *p*<0.0001). C) Representative examples of T1-weighted MRI of control (upper row) and treated (lower row) E98 bearing animals before (pre) and 2–3 minutes after (post) Gd-DTPA injection. [Post-pre]/pre represents subtracted images. Note the complete loss of contrast enhancement in treated animals. Panel D shows H&E staining of sections, corresponding to the slices shown in the MR images. Bars: overviews 2 mm, zoom 200 µm.

**Figure 4:**
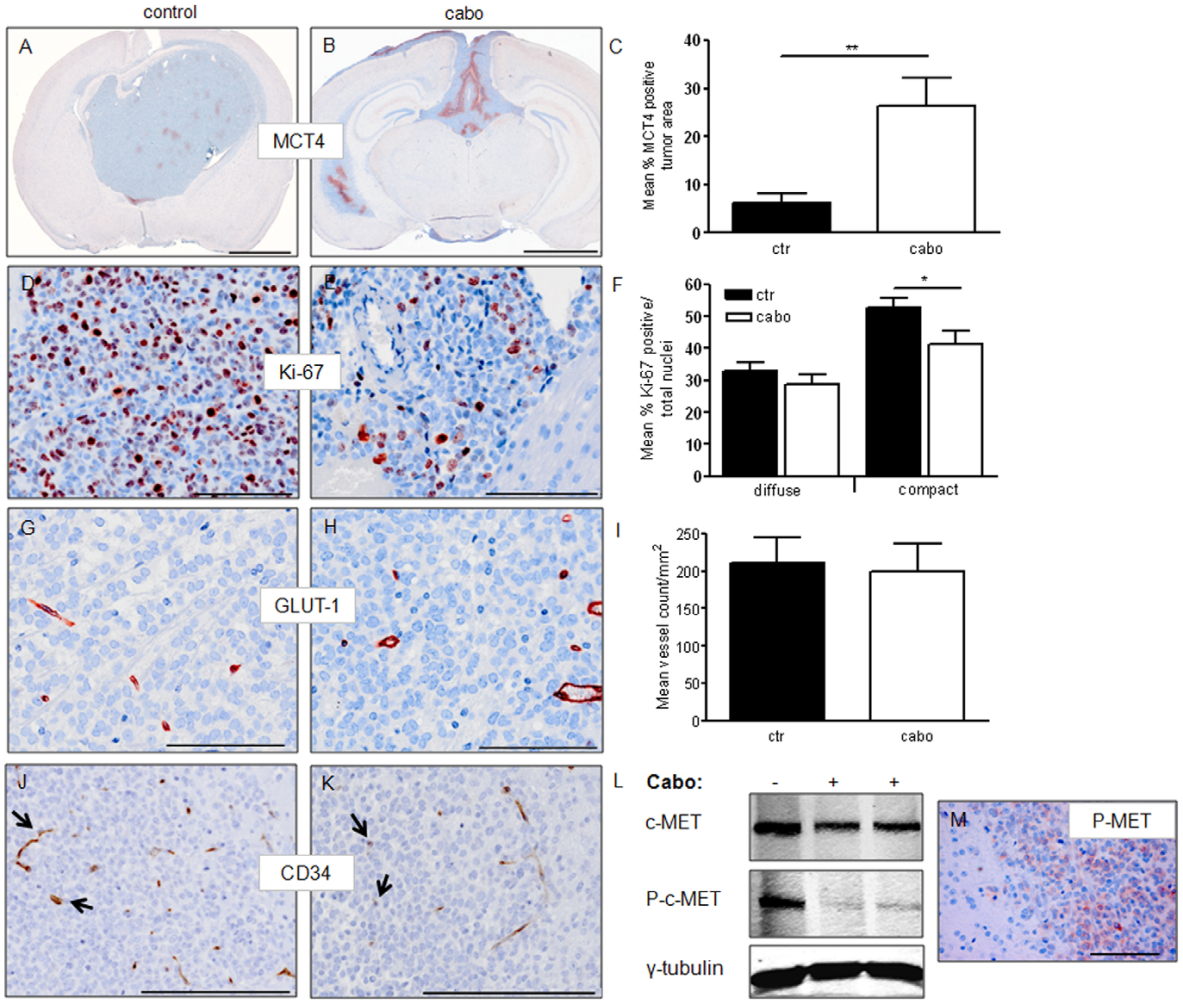
*In vivo* effects of cabozantinib treatment in E98 xenografts. Panels A and B show representative examples of IHC for the hypoxia marker MCT4 in control and cabozantinib-treated tumor bearing animals. Hypoxia in compact tumor regions is significantly increased after treatment (Students *t*-test, p = 0.003, panel C). D and E show examples of Ki67 stainings in compact tumor areas. Proliferation indices were significantly different in these regions (Students *t-*test p = 0.04), but no difference was detected in diffuse tumor areas (panel F). Panels G and H show representative examples of GLUT-1 vessel staining. Automated quantification revealed no differences between vessel densities of diffuse tumor areas in control vs treated mice (I). Numbers of CD34-positive vessels were lower in cabozantinib treated mice (see panels J and K, arrows point at blood vessels), but these data were not quantified because vessels without CD34 expression were also observed in these mice. L: Western blot analysis of protein extracts (50 µg protein/lane), derived from cabozantinib-treated xenografts reveals a substantial, though not complete, reduction of c-MET phosphorylation. As a loading control, γ-tubulin was included. Immunohistochemistry for phospho-c-MET (Y1234/1235) also shows the presence of phosphorylated c-MET in treated animals, as visualized in panel K. Size bars: A–B 2 mm, D–E 100 µm, G, H, J, K 200 µm,

We next subjected mice carrying established orthotopic E98 xenografts, as determined by visibility of edema on T2-weighted MR imaging (see Figure 3A for an example) to treatment with cabozantinib. An initial pilot experiment with 60 mg/kg cabozantinib (n = 3) resulted in a full radiologic response using Gd-DTPA enhanced MRI, similar to our previous observations with bevacizumab, vandetanib and sunitinib [7]–[9]. However, large invasive tumors with hypoxic compact regions (identified by MCT4 expression) remained present after treatment while no signs of hypoxia were seen in the diffuse tumor areas (not shown). There was a non-significant trend towards increased survival (mean survival of 19 days in control vs. 23 days in 60 mg/kg cabozantinib treated animals). A larger group of animals (n = 10) was therefore treated with 100 mg/kg cabozantinib, which did result in significantly prolonged survival compared to control-treated mice (median survival of tumor-bearing control mice was 20 days vs. 32 days for the 100 mg/kg cabozantinib group, log rank test p<0.0001, Figure 3B). All further experiments refer to this group. Prior to sacrifice, mice were subjected to Gd-DTPA-enhanced MRI. Treatment with 100 mg/kg cabozantinib resulted, as expected, again in a complete absence of contrast enhancement (Figure 3C, lower panels) despite clear presence of extensive tumor (H&E staining in figure 3D, note that these sections correspond to the MR scans). Upon 100 mg/kg cabozantinib treatment, tumors generally had converted to a mainly diffuse infiltrative phenotype (see figure 3D, lower panels) similar to our previous findings with vandetanib and sunitinib [8], [9]. In all animals limited areas of compact tumor were present to varying extent and these areas were significantly more hypoxic than in control animals (p = 0.003, see IHC for hypoxia induced monocarboxylate transporter-4 (MCT4, Figure 4A–C) confirming previous observations with other angiogenesis inhibitors [9]. Staining for GLUT-1, another marker for hypoxia, gave similar results (not shown).

Diffuse infiltrative tumor regions did not show signs of hypoxia in control and treated tumors and based on caspase stainings apoptotic cells were very infrequent, also in treated tumors (not shown). Both compact and more invasive tumor areas were analyzed for proliferation differences, based on Ki-67 positivity in IHC. The proliferative fraction did not differ between controls and treated tumors in diffuse infiltrative regions, but proliferation was significantly less in compact tumor regions after treatment (p = 0.04, Figure 4D–F).

GLUT-1 is, except for hypoxic cells, also expressed on brain capillaries and can be utilized as a blood vessel marker in the CNS. Tumor vessel densities were quantified and did not differ in diffuse areas between treatment and placebo groups (Figure 4G–I). Vessel densities in compact areas were difficult to quantify based on GLUT-1 staining because neovasculature often does not express this differentiation marker. Based on CD34 stainings (a marker of activated (neo)vasculature), vessel densities appeared lower in cabozantinib treated animals (compare Figure 4J and K), but as this treatment may also result in a downregulation of CD34 expression on vessels, these data are difficult to interpret in terms of vascular densities. It is important to realize that our studies were not time-matched, making it impossible to compare tumor volumes in treated and control animals.

To investigate whether the remaining diffuse infiltrative tumor might be the result of incomplete c-MET inhibition, we prepared tumor extracts from treated and control animal brains and prepared Western blots. As opposed to our *in vitro* data, even dosing as high as 100 mg/kg cabozantinib (resulting in plasma levels of 30 µM [29]) was not able to fully prevent c-MET phosphorylation (Figure 4L) and this finding was confirmed with IHC for phospho-c-MET (Y1234/1235, Figure 4M).

## Discussion

The proposed functional role of c-MET in tumor cell migration makes this receptor an attractive therapeutic target in c-MET positive glioblastoma. The high expression and activation levels of c-MET in diffuse E98 tumor areas supports the notion that c-MET is actively involved in tumor cell migration [20]. Targeting of c-MET may further be beneficial as signaling from this receptor may induce and maintain the glioblastoma stem cell-like phenotype and therefore resistance to chemotherapy and radiotherapy [35].

Cabozantinib treatment of *in vitro* cultures of E98 cells greatly reduced phosphorylation of AKT and ERK. This was accompanied by a 50% inhibition of cell growth at concentrations as low as 89 nM, which is consistent with the involvement of these signal transducers in PI3K and MAPK signaling. *In vitro* migration assays showed potent effects of cabozantinib on single cell migration, further strengthening a role of c-MET in cell migration as well.

E98 tumors became largely invisible in CE-MRI under cabozantinib therapy, and similar phenomena have been observed in clinical studies, with a radiological response as early as one day after start of therapy [36]. This effect of vascular normalization, rendering capillaries in brain tumors impermeable to MR contrast agents by restoring the blood-brain barrier, is a well known phenomenon which is the result of VEGFR2 inhibition [7], [15], [37]. A reduced vessel density in compact tumor areas in cabozantinib treated mice may also have contributed to some extent to the reduced visibility in CE-MRI. The persistent presence of MRI-invisible tumor confirms our earlier finding that the high blood vessel density in brain can accommodate tumor growth via vessel cooption [14], i.e. without the necessity for neovascularization.

High dose cabozantinib treatment in our study resulted in a significant improvement of survival. Kamoun *et al.* have shown that anti-angiogenic treatment of mice carrying orthotopic glioma may result in improved survival by a sole reduction of edema [38]. In previous experiments, we examined the effects of VEGFR2 inhibition by bevacizumab, vandetanib and sunitinib, alone or in combination. Even combinations of these compounds did not result in improved survival of E98-carrying mice, although there seemed to be a complete blood-brain barrier restoration [9]. Therefore, it is likely that improved survival is the result of additional c-MET targeting. Of note, a phase II trial of cabozantinib for recurrent glioblastoma also revealed clinical activity of this compound [36].

The high efficacy of cabozantinib to E98 cells *in vitro* contrasts with our *in vivo* results. Although the increased survival was unprecedented compared to previously used angiogenesis inhibitors, tumors could still escape therapy via diffuse growth. The exact contribution of c-MET during tumor progression in E98 xenografts is somewhat difficult to assess. In a recent paper an interesting explanation for increased invasiveness of glioblastoma in response to VEGF inhibition has been proposed [20]. These authors demonstrated that c-MET activation is inhibited in glioblastoma cells that also express VEGFR2 in the presence of VEGF. This was suggested to be the result of activation of the VEGFR2-associated protein tyrosine phosphatase PTP1B, resulting in dephosphorylation of its target c-MET. According to this hypothesis, inhibition of VEGF-A releases PTP1B from the multi-receptor complex, unleashing c-MET and resulting in increased diffuse infiltrative tumor growth. This might complicate the use of compounds like cabozantinib, as it may bear antagonistic activities in itself. However, it must be realized that this is only relevant for glioblastomas that are positive for both c-MET and VEGFR2. As E98 tumor cells do not express VEGFR2 this hypothesis is not applicable to this model and other explanations must be found for the escape from therapy.

One such alternative explanation comes from our finding that cabozantinib plasma concentrations of approximately 30 µM [29] did not entirely annihilate c-MET activation whereas *in vitro*, concentrations as low as 0.5 µM sufficed to achieve complete inhibition. Reduced penetration of the drug into the tumor and surrounding tissue, as well as reduced free concentrations of drug due to protein-binding in blood and tissue, may account for the apparent reduction in potency. Pharmacokinetic studies and measurement of steady-state levels in brain would be appropriate.

The difference between the apparent *in vitro* and *in vivo* potency of cabozantinib with respect to c-MET inhibition poses us with an interesting dilemma. We demonstrated in a previous study that the combination of vandetanib and the DNA-alkylating agent temozolomide was less effective than temozolomide alone [8]. This was attributed to vessel normalization and concomitant ‘restoration’ of the blood-brain barrier, resulting in a hampered distribution of the chemotherapeutic agent to the tumor cells. To what extent cabozantinib can pass the blood-brain barrier is not exactly known. If passage would be inefficient, it may be envisioned that a short period of cabozantinib treatment, enough to restore the blood-brain barrier, will result in inhibition of distribution of the compound to tumor cells in later stages of treatment. According to this hypothesis, only during the initial cabozantinib administrations, the compound will reach tumor cells and have anti-migratory and anti-proliferative effects. In later stages of tumor growth, tumor access may be limited by the restored blood-brain barrier, although it is likely that this block is not 100%, given that phospho-c-MET is still significantly reduced in treated tumors. This may indeed explain the significant delay in tumor growth that we observed. In this respect it would be very worthwhile to investigate whether sequential targeting of c-MET and VEGFR2 with monotargeted compounds would be more effective. Such schemes may involve giving intermittent cycles of c-MET inhibitor, followed by VEGFR2 inhibitors, which would effectively result in locking up the c-MET inhibitors in the tumor cell compartment. Such studies are underway in our lab.
